# Modelling vaporised hydrogen peroxide efficacy against mono-species biofilms

**DOI:** 10.1038/s41598-018-30706-0

**Published:** 2018-08-16

**Authors:** F. Watson, C. W. Keevil, S. A. Wilks, J. Chewins

**Affiliations:** 1 Environmental Healthcare Unit, Centre for Biological Sciences, Life Sciences Building, Highfield Campus, University of Southampton, Southampton, SO17 1BJ UK; 2 BIOQUELL (UK) Ltd. 52 Royce Close, West Portway, Andover, SP10 3TS UK

## Abstract

This pilot study investigates a novel approach towards efficacy testing of antimicrobial cleaning agents; focusing primarily on hydrogen peroxide vapour (HPV). Contaminated surfaces are recognised modes of pathogen transmission within healthcare environments and increase the risk of pathogen acquisition in newly admitted patients. Studies have shown these pathogens can survive on surfaces for extended periods of time in spite of cleaning. This resilience is characteristic of biofilm formation and recent publications have identified their presence in hospitals. In this study, biofilm models comprised of multidrug-resistant organisms (MDROs) were generated using a drip flow reactor and exposed to HPV decontamination. The MDROs included *Acinetobacter baumannii*, *Enterococcus faecalis*, *Klebsiella pneumoniae*, *Pseudomonas aeruginosa* and *Staphylococcus aureus*. Upon exposure, samples were periodically removed and enumerated to generate kill curves for each species. Consequently revealing any inherent resistances; such as catalase-producing organisms which expressed reduced susceptibility. Epifluorescence microscopy revealed an abundance of viable and non-viable microcolonies before and after decontamination, respectively. Greater than 6-Log_10_ reduction was achieved within a 100 minutes exposure time. This pilot study puts forward a potential methodology for testing antimicrobial agents against biofilms and supports the efficacy of HPV.

## Introduction

Multi-drug resistant organisms (MDROs) are a growing threat to public health globally^1^. Studies have begun to identify that these MDROs are residing on clinical surfaces in communities known as biofilms^2^. Environmental biofilms are becoming recognised as a source for pathogenesis; acting as a reservoir for infection^3^. By definition a biofilm is a community of microorganisms within a matrix of extracellular polymeric substances (EPS), and which express altered phenotypes in comparison to their planktonic (free-swimming) forms. This altered phenotype is due to the compact nature of the community and is a result of enhanced protein production, gene expression and cell-to-cell communication^4^. The intrinsic properties of a biofilm mean it can be up to 1000x less susceptible to antimicrobial agents, such as antibiotics and disinfectants^5^.

Microbes have been shown to survive for extended periods of time on dry surfaces, for example >5 months for *Acinetobacter sp*., and in a study by Vickery *et al*. biofilm presence was observed on clinical samples over a period of 12 months in spite of routine cleaning of the surfaces using chlorine-based disinfectants^2,6^. It can be suggested that a link exists between biofilm incidence and the difficulties with managing MDROs or hospital-acquired infections (HAIs) in a healthcare setting. Moreover it questions why routine cleaning disinfectants, such as hypochlorites, with proven efficacy using accepted standard tests against planktonic organisms are failing to achieve the desired results^7^.

Hydrogen peroxide vapour (HPV) is a widely accepted technology for the elimination of microbiological contamination on surfaces. As with most decontamination processes the literature used to support its efficacy is generally associated with planktonic organisms deposited onto a dry surface^8–10^. The aim of this pilot study was to assess the efficacious properties of hydrogen peroxide against mono-species biofilms in a dry state; therefore creating a more representative challenge of a contaminated clinical surface.

## Results

**Figure 1:**
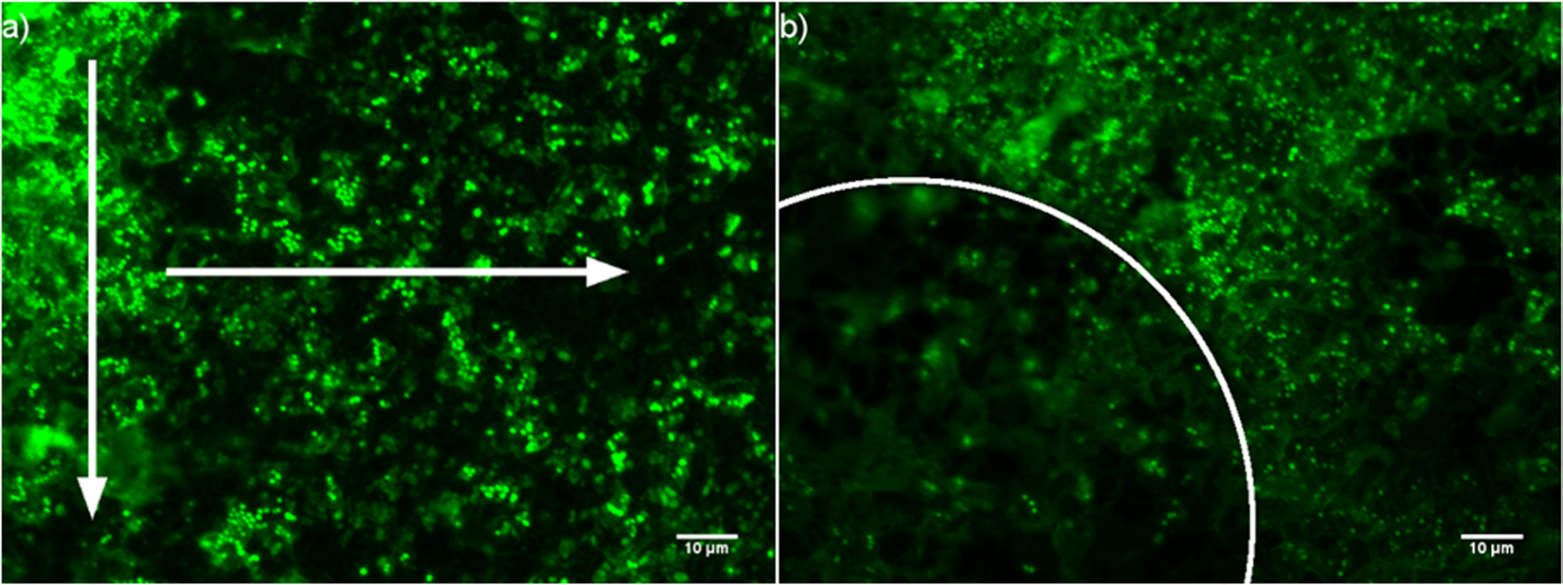
EF micrographs to show the spatial distribution of microcolonies within the biofilm. (**a**) The y-axis arrow depicts the directional flow of the media, whilst the x-axis arrow highlights the decreasing density of microcolonies with a suspected nutrient gradient. This was observed along the entire length of the coupon. (**b**) The arc demonstrates the circular edge of the droplet zone highlighting a boundary in microcolony density and porosity.

The drip flow reactor (DFR) generated confluent bacterial biofilms with distinct structural features, in part due to its unique air-liquid-solid interface and low shear forces^11^. Microcolonies, visualised during epifluorescence (EF) microscopy, were seen to aggregate along the cracks and crevices of the stainless steel coupons. Additionally, the central channel along which nutrient rich media flows saw dense aggregation of microcolonies decreasing in density towards the periphery. A variation in formation, density and distribution of colonies along the coupons axes is inherent of a DFR. The nutrient gradients which form along the longitude and latitude due to the flow and diffusion of the fluid contribute to these variations (Fig. 1a). Moreover, the resultant shear force of the fluid across the surface can influence microcolony formation; seen especially at the droplet zone where the fluid initially strikes. The biofilm appears more porous at this point; a potential consequence of these larger forces. The circular edges that surround this zone were defined by increased microcolony density where the impact forces are lessened (Fig. 1b).

The growth conditions resulted in an average population loading at the end of both the media and desiccation (i.e. zero minutes) phases of 7.15 −Log_10_ colony forming units (CFU) per cm^2^. Notably greater than that expected of clinical surfaces of 5.74-Log_10_^7^. Table 1 highlights the average Log_10_ loadings and repeatability in standard deviation (SD) per species. The Log_10_ loadings for each species between these phases deviated ≤9.32%; with the exception of *Enterococcus faecalis* which demonstrated a deviation of 29.85%. Nosocomial pathogens have previously been shown to survive on dry surfaces for extended periods of time including *E. faecalis*^12^. The reduced population of viable bacteria in *E. faecalis* therefore suggests in this study there was an increased susceptibility to drying. Nonetheless, the results demonstrate the tolerance expressed by bacteria in biofilms during desiccation.

**Table 1:** Statistical representation of the control coupons for each of the five bacterial species tested. An estimated standard deviation of 0.23 was expected for log loadings post the media phase^23^.

Test Species	Post media phase/Log_10_ CFU cm^−2^	SD	Post desiccation phase/Log_10_ CFU cm^−2^	SD
*Acinetobacter baumannii*	6.72	0.31	7.11	0.13
*Enterococcus faecalis*	7.89	0.02	6.08	1.63
*Klebsiella pneumoniae*	8.09	0.14	8.27	0.95
*Pseudomonas aeruginosa*	6.19	0.04	6.69	0.71
*Staphylococcus aureus*	6.89	0.29	7.59	0.86

**Figure 2:**
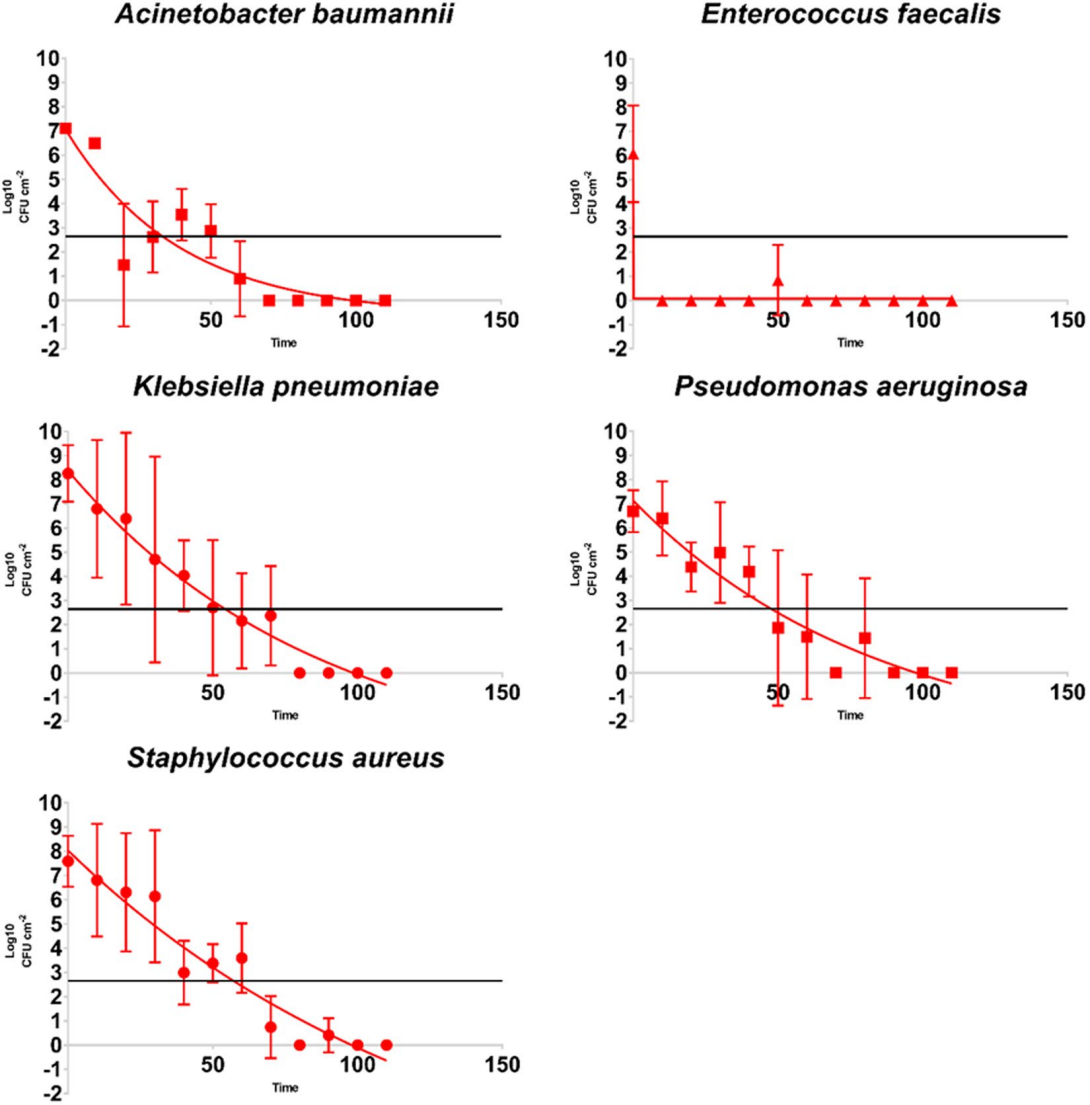
Kill curves of mono-species biofilms in a dry state. The limit of detection is indicated by the solid horizontal line.

HPV exposure achieved >6-Log_10_ CFU cm^−2^ reduction across all five species with complete inactivation occurring within 100 minutes exposure time (Fig. 2). Greater than 90% of this reduction occurred within the initial 50 minutes.

The kill curves obtained during exposure to HPV exhibit noticeable variation in Log_10_ cfu loadings per time point. We speculate this to be exclusion of the HPV molecules from the surface and/or base of the biofilm. Hydrogen peroxide relies on direct contact to elicit kill, thus bacteria within the biofilm may have evaded or been protected from oxidation. A dense matrix can thwart the diffusion of antimicrobial agents to the basal bacteria^4^. Possibly, but less likely, oxidative stress or DNA damage may have driven the bacteria into a dormant state and, as a consequence, the population can remain viable but are no longer culturable with routine detection methods^13^.

**Figure 3:**
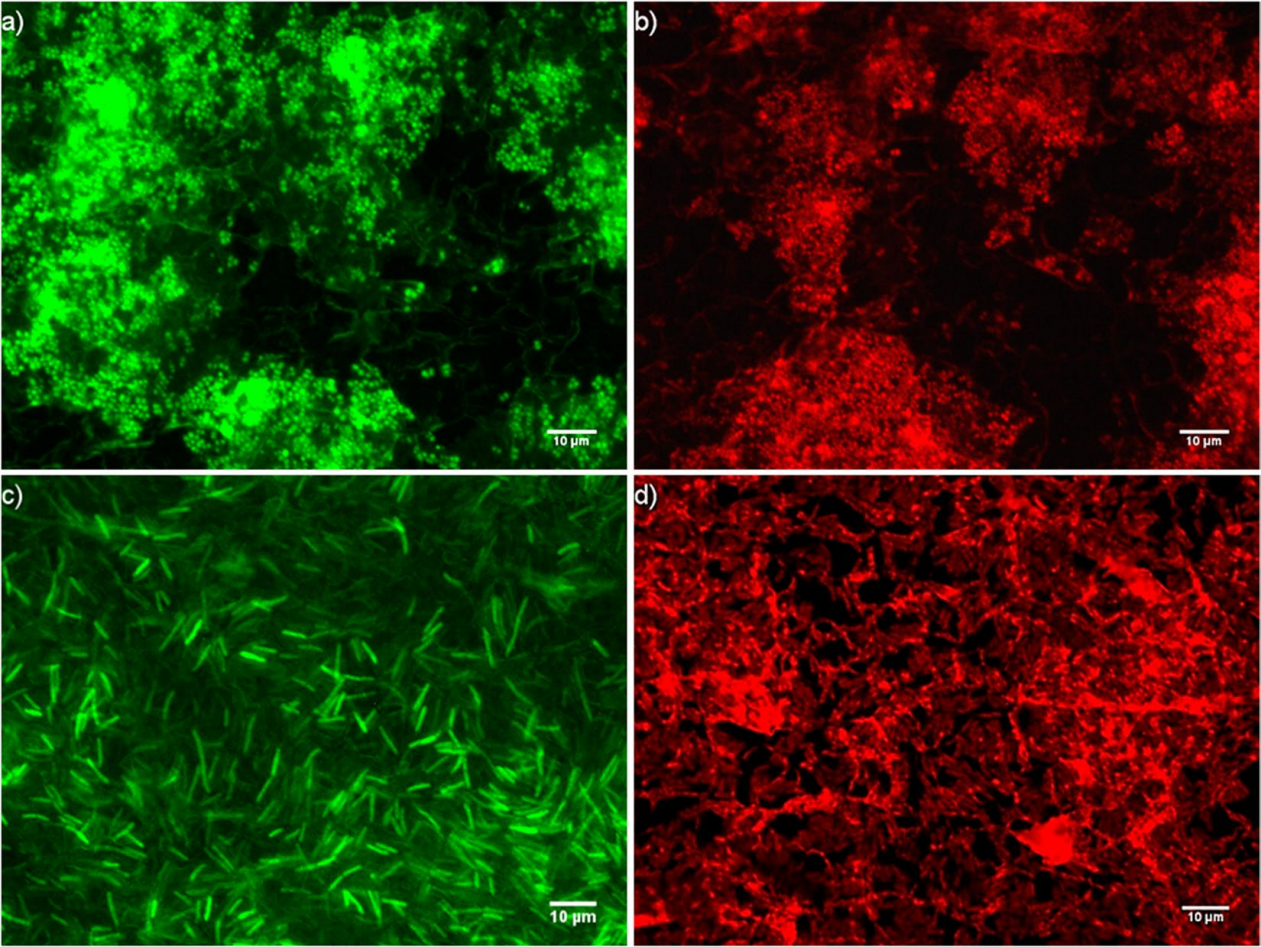
EF micrographs of *Staphylococcus aureus* (Top) and *Klebsiella pneumoniae* (Bottom) biofilms stained with SYTO-9 (**a**,**c**) and propidium iodide (**b**,**d**).

EF microscopy revealed an abundance of ‘live’ (intact membraned cells, stained with SYTO-9) bacteria forming microcolonies throughout the biofilm model prior HPV exposure (Fig. 3a,c). This was complemented by micrographs of ‘dead’ (compromised membraned cells, stained with propidium iodide) bacteria in microcolonies afterwards (Fig. 3b,d). A visible change to microcolony formation can be seen between the control and processed coupons; with those exposed to HPV showing signs of more compact clustering and fragmented formations. Equivalent results were observed across all species (data not shown).

## Discussion

Biofilms express an increased virulence as well as a phenotypic resistance to antimicrobial agents, all of which will contribute to higher risk of pathogen acquisition in patients. About 65% of nosocomial infections are believed to involve a biofilm^14^. Although these are often reported in relation to medical implants or indwelling devices, such as catheters, studies are beginning to evaluate environmental surface contamination and the role they may play in hospital infection rates. Biofilm formation is often only associated with a damp or wet surface; however, a study from Hu *et al*. disproves this and postulates room humidity and/or moist microclimates in close proximity to patients are able to sustain biofilm forming species^15^.

The DFR used in this pilot study generated a highly confluent biofilm model using five strains of the six *ESKAPE* pathogens; a term coined by the Infectious Diseases Society of America, encapsulating the top antibiotic resistant species across the globe^16^. The DFR model was chosen for its unique air-liquid-solid interface and gravitionally driven low shear forces. We percieved these conditions, in combination with the decissation model, to more-closely ressemble conditions found on clincial surfaces. Buckingham-Meyer *et al*. emphasises the importance of growing biofilms for efficacy studies under fluid flow conditions similar to that of the environment in question^17^. The growth media used in the DFR acts not only as a mechanical force but as a medium for exchanging metabolites and nutrients; the result of which significantly influences microcolony and niche formation, structuring and phenotypical behaviours. During EF microscopy a gradient in microcolony density and distribution, both latitudinal and longitudinal, can be observed – plausibly linked to a combination of these flow conditions^18^.

Biofilm communities possess an inherent phenotypical response to desiccation and as a result are largely hygroscopic and slow to dry out. As a result the EPS matrix of a biofilm will become more concentrated during periods of desiccation through heightened levels of EPS production and increased number of non-specific binding sites^4,19^. This response will differ amongst species. Furthermore, we would expect this to have an adverse effect upon antimicrobial agents. This could be used to explain the variances seen here in our viability counts.

The efficacy of HPV has been demonstrated against an array of bacterial, viral and fungal species, but our study is believed to be the first to investigate its efficacy against biofilms. We were able to report the ability of HPV to achieve >6-Log_10_ reduction of all species within 100 minutes exposure time as presented within the kill curves in a large enclosure/room based scenario. The presence of catalase in catalase-producing organisms, such as *Staphylococcus aureus*, has been shown to reduce the efficacy of hydrogen peroxide; although it does not appear to have influenced the results in this study^20^. From these results we can speculate the levels of susceptibility amongst strains of nosocomial bacteria in biofilm.

In addition to the log reductions achieved, EF microscopy indicated a noticeable disruption in microcolony formation and assumingly EPS in close proximity; as a result of the oxidative damage caused by the hydrogen peroxide molecules. The biocidal properties of hydrogen peroxide are well founded and utilised globally; this form of decontamination is not target specific and will readily attack proteins, lipids and nucleic acids alike^21^. EF micrographs of HPV exposed biofilm samples demonstrate microcolonies to be more tightly packed and a ‘flaking’ or ‘crusting’ of the biofilm surface. The matrix of EPS surrounding a biofilm community is comprised of polysaccharides, liposaccharides, nucleic acids and proteins; all of which are targets for hydrogen peroxide molecules^4^. The result of our investigation suggests HPV causes massive disruption in both microcolonies and EPS matrix within the biofilm community.

## Conclusion

This pilot study demonstrates a novel approach to efficacy testing for hospital disinfectants; in this instance HPV upon mono-species biofilms. This model showed that viable strains of high concentration MDROs in biofilms subjected to periods of desiccation are inactivated upon exposure to HPV. As with all naturally occurring ecosystems, we readily expect clinical biofilms to comprise of multiple species – not just bacterial. Therefore these results need to be confirmed with mixed species biofilms that are representative of the clinical environment. We postulate this to be accompanied by an increased resistance to antimicrobials as a result of synergy within the community.

Microscopy of the biofilm models confirm the unique characteristics of a DFR, caused by nutrient gradients and shear forces. Based upon previous publications this model is considered to have a low Reynold’s number (12–20) with slow laminar flow^17^. As a result, the biofilm will have a weaker adhesion to hard surfaces when compared to high shear force models such as CDC reactors. Therefore, we acknowledge this model alone may not be representative of all clinical surfaces such as those subject to high mechanical stresses associated with cleaning and/or wiping. Furthermore, we recognise there is a lack in clinical biofilm evidence which would allow a comparison of phenotypical traits; therefore disputing the application of a DFR for use in hospital disinfectant testing. It is hypothesized that surface condensation and/or relative humidity within the clinical environment is responsible for initiating biofilm formation^2^. This is believed to be in combination with pathogen transmission from patients. A greater number of analytical studies on clinical surface biofilms is necessary to enable these phenotypical traits to be distinguished. Future investigations could benefit from understanding the similarity between this *in vitro* model and *in situ* clinical microbiomes. This would benefit from incorporating the impact of desiccation which may enhance resistance to mechanical removal.

Biofilm models will often utilise simple, or derivatives of, commonly purchased enriched growth broth media. It would be beneficial for further studies to focus on emulating the *in situ* availability of nutrients within solutions indicative of a healthcare environment; such as cleaning detergents and bodily fluids. This work would integrate cyclic exposure of these solutions as, for example, this would more closely represent the periodic cleaning routine of hard surfaces. Repeated rehydration and dehydration of a biofilm is expected to increase EPS production and thus its tolerance to disinfectants^22^. We identify this limitation within our desiccation model and will concentrate future work on replicating the variable conditions associated with hospital cleaning procedures.

The biofilm samples used in the study contained an average of 7.15-Log_10_ CFU cm^−2^ noticeably high for the level of contamination found on dry hospital surfaces, which ranges from 2.62 to 7.20-Log_10_^7^. We observed a limitation in the results due to the lack of a large number of replicate coupons per measurement point during this study. We identified within our results a notable variance demonstrated by the SD during HPV exposure in comparison to those observed post media phase. There are many contributing factors at this stage including strains of bacteria, environmental conditions and nutrients retention. Nonetheless, this pilot study does highlight the efficacious capabilities of HPV in spite of the greater challenge posed by an increased population size. The abundance of nutrients was key to the population expansion seen on our coupons. Previous authors of biofilm models, which share similarities to clinical samples, have shown a negative correlation in EPS density with the effectiveness of disinfectants^22^. Future work will concentrate efforts to include biomass analysis in conjunction with viability counts; as this will influence the biofilms ability to retain moisture and heightened tolerance to non-specific antimicrobial agents.

A greater number of clinical studies on biofilms are required across a diverse range of healthcare surfaces before they can be regarded as common within this type of environment. If found true, it may provide a vital explanation to the difficulties experienced when tackling surface contamination, increasing levels of HAIs, and why orthodox cleaning programmes fail to perform.

## Methodology

### Bacterial strains

The bacterial strains used in this study possessed genes capable of expressing drug resistance mechanisms to the extended-spectrum beta-lactamase antibiotic group. These strains were used: *Acinetobacter baumannii* (NCTC: 13301), *Enterococcus faecalis* (NCTC: 13379), *Klebsiella pneumoniae* (NCTC: 13438), *Pseudomonas aeruginosa* (ATCC: 15692) and *Staphylococcus aureus* (NCTC: 11939). All the species used were chosen for their ability to form biofilms and known persistence on healthcare surfaces.

### Inoculum preparation

All bacterial strains were sub-cultured into 10 mL of tryptic soya broth (TSB) (Sigma Aldrich) overnight at 37 °C. The number of CFU per mL of bacterial suspension was quantified using serial dilutions and incubation on tryptic soya agar (TSA) (Sigma Aldrich) for 24 hours at 37 °C.

### Biofilm model

The biofilms were generated in a drip flow reactor (DFR) (BioSurface Technologies, Bozeman, MT) assembled as per ASTM E2647-13 using 316 stainless steel coupons as the substrate^23^. The coupons were inoculated with 1 mL of culture inoculum at a population of 7 to 8-log_10_ CFU and incubated for 6 hours at room temperature (≈20 °C), referred to here as the batch phase. The reactor was then tilted to a 10° angle to allow sufficient drainage of waste and shear force across the coupon surface. A sterile supply of TSB solution was initiated by means of a 6 channel peristaltic pump (Cole Palmer™) at a flow rate of ≈0.9 mL min^−1^ per channel for 36–48 hours at room temperature, referred to here as the media phase. The TSB medium concentration used is previously described for different bacterial strains: *P. aeruginosa* and *K. pneumoniae* = 1%, *E. faecalis*, *A. baumannii* and *S. aureus* = 5%^24^.

### Desiccation model

The efficacy of hydrogen peroxide vapour technology was measured against biofilm in a dry state. A dry state biofilm uses a previously described method for biofilm dehydration by means of an aquatic air pump (Hailea) passing room air, via a 0.2 µm in-line filter (Fisher Scientific™) across the media surface at 3 L min^−1^ in a 0.01 m^3^ container for 48 to 66 hours; referred to here as the drying phase^22^. All biofilm coupons were exposed to a single dehydration cycle.

### Hydrogen peroxide vapour exposure cycle

A Bioquell fixed system hydrogen peroxide vapour generator (Bioquell UK, Andover) was mounted on the wall of a ≈30 m^3^ test enclosure. The enclosure was fitted with glove and transfer ports to enable manipulation and removal of biological samples. The enclosure’s environment was maintained at 20 ± 5 °C and 50 ± 10% RH for the start of each experiment. The cycle consisted of an injection phase whereby 270 g of 35% w/w hydrogen peroxide was injected, followed by a 120 minute dwell period of zero injection.

An appropriate number of biological samples (usually 10–12) were placed into an apparatus that minimalised the occlusion of biofilm matter from HPV and were periodically removed from the HPV environment at the start of gassing.

### Quantification of coupon population

To remove any residual hydrogen peroxide vapour from the surface the samples were immediately transferred and exposed in a Class 2 Biological Safety Cabinet, allowing rapid aeration of the coupon surface for up to 5 minutes before being submerged in Phosphate Buffer Solution (Oxoid™). The Log_10_ CFU per cm^2^ of each coupon was quantified using a method previously described in ASTM E2647-13^23^. Coupons which produced counts below the limit of detection were re-suspended in TSB and incubated overnight at 37 °C to detect viable cultures; lack of growth was considered complete inactivation.

### Quantification of biofilm colonies

Samples were stained with LIVE/DEAD BacLight™ bacterial viability kits (Invitrogen); this included both SYTO-9 (green) and propidium iodide (red). EF microscopy was used to visualise ‘live’ and ‘dead’ bacterial cells, as previously described^25^. The datasets generated and analysed during this study are available from the corresponding author upon reasonable request.

### Experimental design

In each experiment a single bacterial species was generated and tested against HPV. The enclosure was maintained within the parameters as stated previously for temperature and room humidity so that kill curves could be compared with minimal variability due to external factors. For each species of bacteria the experiment was repeated 3 times for a total of 15 experimental runs. The same technique and technician was used to conduct all experiments. Coupons were randomly selected for each time point.

### Statistical analysis

The log loadings recorded for each coupon were transformed to Log_10_ CFU cm^−2^ and all statistical calculations were performed using these values. The average log loadings and standard deviation for coupons post media and desiccation phase were calculated for each species. The deviation between these phases was calculated as percentage difference. The average and standard deviation for each time point was calculated for each species and plotted onto a graph to visualise their respective kill curve.
